# Knowledge about juvenile idiopathic arthritis-associated uveitis: more frequent reminders are associated with higher patient and family uveitis knowledge

**DOI:** 10.1186/s12969-021-00639-6

**Published:** 2021-10-02

**Authors:** Ashley M. Cooper, Elaine R. Flanagan, Tova Ronis, Baruch Goldberg, Ashley K. Sherman, Chelsey Smith, Gary N. Holland, for the CARRA Uveitis Workgroup

**Affiliations:** 1 Children’s Mercy Kansas City, 2401 Gillham Rd, Kansas City, MO 64108 USA; 2 University of Missouri Kansas City School of Medicine, Kansas City, MO USA; 3 Emory University School of Medicine, Atlanta, GA USA; 4 Children’s National Hospital and George Washington University, Washington, DC USA; 5 UCLA Stein Eye Institute and the Department of Ophthalmology, David Geffen School of Medicine at UCLA, University of California Los Angeles, Los Angeles, CA USA

**Keywords:** Uveitis, Juvenile arthritis, Patient education

## Abstract

**Background:**

Chronic anterior uveitis is a sight-threatening complication of juvenile idiopathic arthritis (JIA) and a primary contributor to long-term morbidity in people with JIA. Levels of knowledge about uveitis among JIA patients and their parents are unknown. A survey of JIA patients and parents was conducted to assess knowledge about uveitis complications and recommended screening.

**Methods:**

A survey was developed consisting of six demographic questions, six arthritis/uveitis history questions, and nine uveitis knowledge questions. The survey was administered to JIA patients age 14 and older and parents of patients with JIA at three pediatric rheumatology practices and online through the Patients, Advocates, and Rheumatology Teams Network for Research and Service (PARTNERS) network. ANOVA, chi-square and Fisher’s exact tests were used to look for relationships between survey questions and demographic variables.

**Results:**

Thirty-three patients and 111 parents completed the survey. Overall, 17.4% reported a history of uveitis, and 89.6% had heard of uveitis. The mean composite knowledge score was 6.46 ± 2.6 out of 9. Patients and parents with a history of uveitis had higher composite knowledge scores than their counterparts without a uveitis history (*p* = 0.01 and *p* < 0.01, respectively). Parents whose rheumatologist reminded them about eye exams at every visit had higher knowledge of the risk of blindness (*p* = 0.04), the risk for uveitis when arthritis is controlled (*p* = 0.02), the need for ongoing eye exams when off of medications (*p* = 0.01), and had a higher overall score (*p* = 0.02) than those who were reminded at some visits or not at all.

**Conclusions:**

JIA patients and parents report variable levels of knowledge regarding uveitis complications and recommended screening. Frequent discussion between the rheumatology provider and family about uveitis screening is associated with higher uveitis knowledge. Incorporating detailed and frequent education about uveitis into rheumatology clinic appointments may improve early uveitis detection and visual outcomes.

**Supplementary Information:**

The online version contains supplementary material available at 10.1186/s12969-021-00639-6.

## Background

Juvenile idiopathic arthritis (JIA) is the most common rheumatic disease of childhood. In addition to joint inflammation, 10–20% of children with JIA develop chronic anterior uveitis [1, 2]. Although JIA-associated uveitis is often asymptomatic early in the disease course, it can lead to visual compromise through the development of cataracts, glaucoma, macular edema, band keratopathy, and inflammatory membranes in up to 70% of patients [3–7]. Screening for uveitis consists of measuring immune cells and protein (“flare”) in aqueous humor of the anterior chamber of the eye, as seen by slit lamp biomicroscopic exam per the Standardized Uveitis Nomenclature (SUN) criteria and evaluating visual acuity and ocular pressure [8]. The current screening algorithm developed for the American Academy of Pediatrics (AAP) recommends screening patients every 3–12 months depending on known risk factors: arthritis subtype, antinuclear antibody (ANA) status, age of arthritis onset, and time since arthritis onset [9]. In 2019, the American College of Rheumatology published guidelines that included similar uveitis screening intervals for JIA patients and specified screening intervals for JIA-associated uveitis patients during periods of disease control and medication tapering [10].

Identification of uveitis through screening before symptoms of vision loss are clinically apparent is critical for early treatment and prevention of complications. There is minimal information in the published literature about rates of adherence to recommended eye screening among patients with JIA. Ballenger, et al. examined barriers to uveitis screening at a single center, where 37% of patients with JIA were nonadherent to the recommended screening interval and over one-fourth of patients were not familiar with the recommended frequency of eye exams [11]. A detailed assessment of patient knowledge about the complications of uveitis and the exam components which are necessary to adequately screen for uveitis was not included in that study.

The Childhood Arthritis and Rheumatology Research Alliance (CARRA) Uveitis Workgroup is comprised of pediatric rheumatologists and ophthalmologists with expertise or special interest in childhood uveitis. The clinical experience of the workgroup suggested that JIA patients and parents have poor understanding of potential complications of uveitis, as well as timing and required components of uveitis screening exams. The goal of this survey was to assess JIA patient and parent knowledge about uveitis complications and screening recommendations.

## Methods

In this survey of uveitis knowledge, patients and parents of patients with JIA were recruited from pediatric rheumatology clinics at three geographically-diverse academic centers (Children’s Mercy Kansas City, Children’s National Hospital, Washington, DC, and Children’s Healthcare of Atlanta/Emory University), and online through the PARTNERS (**P**atients, **A**dvocates and **R**esearch **T**eams **Ne**twork for **R**esearch and **S**ervice) collaborative, a patient-powered research network. Respondent inclusion criteria were the following: adolescents and young adults with JIA (age 14 or older) and parents of JIA patients of any age, who could complete a survey in English.

This study was approved by the Institutional Review Boards of the participating sites and documentation of consent/assent was waived.

### Survey design

Patients and parents of patients with JIA were approached to complete the uveitis survey during regularly scheduled pediatric rheumatology clinic visits or completed the survey online through PARTNERS. For recruitment in clinics, only one participant was recruited from each family (an age-eligible patient or a parent). Participants completed a 21-item survey developed by the CARRA Uveitis Workgroup to assess respondent knowledge about uveitis risks, complications, and screening recommendations and procedures. The survey included six demographic questions, six arthritis/uveitis history questions, and nine uveitis knowledge questions. Additional file 1 shows the detailed survey content [see additional file 1] Knowledge questions addressed issues such as potential development of cataracts, glaucoma, and blindness, as well as participant knowledge of the required components of a full ophthalmologic screening visit for uveitis: slit lamp biomicroscopy and intraocular pressure determination. The survey was pilot tested by several parents of JIA patients at one of the participating sites. Feedback from this pilot testing was used to improve the survey design prior to initiating recruitment. Study data were collected anonymously and managed using REDCap (Research Electronic Data Capture) electronic data capture tools hosted at Children’s Mercy Kansas City [12, 13]. REDCap is a secure, web-based software platform designed to support data capture for research studies, providing 1) an intuitive interface for validated data capture; 2) audit trails for tracking data manipulation and export procedures; 3) automated export procedures for seamless data downloads to common statistical packages; and 4) procedures for data integration and interoperability with external sources. As a composite measure, an overall knowledge score was calculated by summing the number of correct individual knowledge questions resulting in a range of 0–9 (Table 1).

**Table 1:** Uveitis knowledge assessment and composite score

Question	Score
1. Have you heard of uveitis (an eye disease that can happen in people with arthritis)?	__/ 1
2. Did you know that uveitis can cause cataracts (clouding of the lens)?	__/ 1
3. Did you know that uveitis can cause glaucoma (eye damage from high pressure)?	__/ 1
4. Did you know that uveitis can cause blindness?	__/ 1
5. Did you know that children with arthritis can get uveitis even when their arthritis is controlled?	__/ 1
6. Did you know that children with arthritis need eye screening even if they have no eye symptoms?	__/ 1
7. Did you know that children with arthritis need eye exams even if they are no longer on medications for arthritis?	__/ 1
8. Did you know that a slit lamp is needed for a full eye exam for uveitis? (picture was shown)	__/ 1
9. Did you know that a pressure check is needed for a full exam for uveitis? (pictures of instruments used to check pressure were shown)	__/ 1
Composite Score	__/ 9

Descriptive statistics such as means, standard deviations, and proportions were used to summarize the data. Associations between demographic or historical features and individual knowledge questions or composite scores were analyzed using chi-square, the Fisher exact, t-tests or ANOVA. *P* values less than 0.05 were considered significant. SAS, version 9.4 (SAS Institute Inc., Cary, NC), was used for all analyses.

## Results

Thirty-three patients and 111 parents completed the survey in clinic or online through PARTNERS. There was one missing response to 4 different questions, otherwise the data was complete. Demographic data are summarized in Table 2, stratified by identity (patient or parent) and location of survey completion (clinic or online). Respondents from all groups were predominantly female (126/144, 87.5% overall) and Caucasian (123/144, 85.4% overall) with a relatively high educational level (62/144, 43.1% college graduates and an additional 36/144, 25% with post-graduate education).

**Table 2:** Demographic information of patient and parent respondents stratified by location of survey completion

	Patients		Parents	
Demographics	Clinic (*n* = 21)	Online (*n* = 12)	Clinic (*n* = 47)	Online (*n* = 64)
Age, median (IQR)	17 (14–19)	26 (23–39.50)	38.5 (36–44)	42 (37–48)
Female, n (%)	17 (81)	11 (91.7)	38 (80.9)	60 (93.8)
Race/Ethnicity				
African American	1 (4.8)	0	3 (6.4)	1 (1.6)
Asian	0	0	4 (8.5)	1 (1.6)
Hispanic	1 (4.8)	2 (16.7)	2 (4.3)	1 (1.6)
Caucasian	18 (85.7)	10 (83.3)	38 (80.9)	57 (89.1)
Other	1 (4.8)	0	0	4 (6.3)
Geographic Location				
Midwest	5 (23.8)	3 (25)	22 (46.8)	23 (35.9)
Northeast	3 (14.3)	2 (16.7)	7 (14.9)	23 (35.9)
Southeast	13 (61.9)	3 (25)	18 (38.3)	10 (15.6)
Other	0	4 (33.3)	0	8 (12.5)
Educational Level				
Some HS/HS grad	4 (19.1)	0	5 (10.6)	2 (3.1)
Some college/trade	7 (33.3)	3 (25)	12 (25.5)	13 (20.3)
College grad	6 (28.6)	5 (41.7)	22 (46.8)	29 (45.3)
Postgrad	4 (19.1)	4 (33.3)	8 (17)	20 (31.3)

A total of 128 respondents (88.9%) reported that they/their child is treated for JIA by pediatric rheumatology, 13 (9%) by adult rheumatology, 2 (1.4%) by a primary care provider, and 1 (1.4%) by “other” (comments indicate ophthalmology in partnership with pediatric rheumatology). Among all respondents, 143 (99.3%) had at least one eye exam and 25 (17.4%) reported a history of uveitis.

Among all participants, 89.6% had heard of uveitis, though knowledge of potential uveitis complications and screening recommendations were lower in all groups (Table 3). The mean composite score was 6.46 (SD 2.6) out of 9. Parent respondents who completed the survey online through PARTNERS had a higher composite score (mean 7.22) than parents completing the survey in clinic or patients completing the survey through PARTNERS or in clinic (means of 5.96, 5.92, 5.57, respectively) (*p* = 0.02, although no pairwise differences were significant).

**Table 3:** Results of individual uveitis knowledge survey questions and composite scores

Positive Responses to Knowledge Questions (%)					
Question (abbreviated)	Patients		Parents		Total
	Clinic (*n* = 21)	Online (*n* = 12)	Clinic (*n* = 47)	Online (*n* = 64)	
Have you heard of uveitis?	71.4	100	91.5	92.2	89.6
Did you know uveitis can cause cataracts?	42.9	50	38.3	64.1	51.4
Did you know uveitis can cause glaucoma?	52.4	41.7	46.8	73.4	59
Did you know uveitis can cause blindness?	66.7	83.3	68.1	89.1	78.5
Did you know uveitis can occur when JIA is controlled?	47.6	66.7	63	82.8	69.9
Did you know eye exams are needed even if no eye symptoms?	71.4	83.3	93.6	92.2	88.9
Did you know eye exams are needed even if off arthritis medications?	57.1	58.3	68.1	78.1	70.1
Did you know a slit lamp is needed for screening?	81	50	76.6	82.8	77.8
Did you know a pressure check is needed for screening?	66.7	58.3	55.3	67.2	62.5
Composite Score, mean (sd)	5.57 (3.3)	5.92 (2.2)	5.96 (2.3)	7.22 (2.6)	6.46 (2.6)

Patients with a history of uveitis had higher composite knowledge scores than those without a uveitis history (8.3 vs. 5, p = 0.01). The same was true for parents who reported a history of uveitis in their child (8.6 vs. 6.3, *p* < 0.01). More parents who reported that their rheumatologist reminded them about eye exams at every visit were aware of: the risk of blindness (85.5% vs. 68.6%, *p* = 0.04), the risk for uveitis even when arthritis is controlled (81.3% vs. 60%, *p* = 0.02), and the need for ongoing eye exams when off medications (81.6% vs. 57.1%, *p* = 0.01) than those who received occasional or no reminders from their rheumatologist. They also had a higher overall knowledge score (mean 7.1 vs. 5.9, *p* = 0.02). Differences in knowledge related to frequency of eye exam reminders did not reach statistical significance in the patient group. There was a trend toward higher composite scores among college graduates when compared to non-college graduates, although the difference did not reach statistical significance. (mean 6.7 vs. 5.9, *p* = 0.07).

## Discussion

Uveitis is a major source of morbidity in JIA patients. Regular screening is required to detect JIA-associated uveitis during the asymptomatic phase, allow timely treatment, and prevent ocular complications. JIA patients receive multi-disciplinary care and parents and patients are a vital bridge between providers. It is essential that they are informed about uveitis risks and necessary screening to function optimally as advocates for their health.

We found no prior articles describing a similar multicenter assessment of uveitis knowledge base in JIA patients on a computerized literature search using PubMed. In this geographically diverse cohort, we demonstrated that knowledge about uveitis screening recommendations, eye exam components, and potential uveitis complications is variable among JIA patients and their parents. Patients and parents of patients with a history of uveitis had higher overall knowledge of uveitis. This finding is not surprising as patients who have been diagnosed with uveitis would likely have discussed the uveitis diagnosis in more detail with their providers, attended more frequent eye exams, and discussed or even experienced complications of uveitis, such as a cataract or glaucoma.

The other factor associated with increased uveitis knowledge among JIA patients and parents was receiving reminders about eye exams at every rheumatology visit. In addition to overall knowledge, reminders were specifically associated with improved understanding of the risks of uveitis when arthritis is controlled and when off of arthritis medications. Understanding of these concepts is crucial for parents and young adult patients to remain vigilant about routine eye exams even during periods of inactive arthritis to promote long term ocular health.

This project was initially pursued to assess the accuracy of the project team’s anecdotal experience that uveitis knowledge is suboptimal in JIA patients and parents. The question regarding eye exam reminders was included in this survey to assess whether discussions about uveitis in the rheumatology office had measurable impact on knowledge base. The findings of this study suggest that regular reminders about eye exams by rheumatologists affect overall uveitis knowledge. Given the long-term therapeutic relationships between JIA families and their rheumatology providers, the rheumatology clinic may be an optimal setting for a targeted intervention to further increase uveitis knowledge and adherence with ophthalmology exams.

There were several limitations of this study. The respondent population lacked diversity with respect to gender and ethnicity, though as several subtypes of JIA are more prevalent in females and Caucasians, this finding is not surprising. Though there was geographic diversity among respondents, the Northwest and Southwest were underrepresented. The goal of including patients from across the United States was to capture practice pattern variation across different rheumatology centers, which was likely achieved with this respondent group.

Several limitations may have contributed to an over-estimate of uveitis knowledge in this study cohort as compared to the larger JIA population. Survey questions were worded using the structure “Did you know” followed by a true statement to avoid risk to participants of being misinformed about uveitis as a result of study participation. This approach may have introduced bias leading to over-reporting of uveitis knowledge. The study population was limited to those who could complete the survey in English. Patients and families with limited English proficiency may face additional barriers to gaining knowledge about JIA-associated uveitis as compared to our study population. As patients and parents with higher levels of education were over-represented, the level of uveitis knowledge and understanding may not be fully generalizable to the overall JIA population. The skewed educational background may over-estimate uveitis knowledge among the larger JIA population rather than under-estimating it. This effect was suggested by a trend towards higher composite scores in the college graduates, though the difference did not reach statistical significance. Similarly, respondents from PARTNERS, who are highly engaged advocates for JIA research and care, may be more knowledgeable about uveitis than most JIA families. Some of the patient participants recruited through PARTNERS are now adults, raising the possibility of recall bias.

Recruiting participants from PARTNERS was extremely valuable, allowing inclusion of highly motivated patients and parents from more sites; however, a limitation of this approach was the inability to definitively verify the diagnosis of JIA in the PARTNERS participants. Finally, it was impossible to determine if the respondents were up to date on their eye exams without access to multiple clinical characteristics that the uveitis screening recommendations are based upon: JIA subtype, age at diagnosis, ANA status, and time since diagnosis.

## Conclusions

Patients and parents of patients with JIA were aware of uveitis, but the depth of knowledge varied. Factors associated with a higher knowledge about uveitis complications and screening recommendations were a personal history of uveitis and eye exam reminders at every rheumatology visit. Incorporating standardized and frequent education about uveitis into rheumatology clinic appointments may be an effective strategy to improve knowledge about uveitis and subsequently visual outcomes in JIA patients, as early diagnosis and treatment is critical for retention of vision. Future directions for this work include development and testing of standardized patient education tools.

## Supplementary Information

**Additional file 1.** Uveitis Education Survey Content. This document shows the detailed content of the survey utilized in this study and demonstrates the branching logic utilized to deliver appropriately worded versions of survey questions to patients versus parents.

## Data Availability

The datasets used and/or analyzed during the current study are available from the corresponding author on reasonable request.

## Abbreviations

JIA: juvenile idiopathic arthritis
PARTNERS: Patients, Advocates, and Rheumatology Teams Network for Research and Service
SUN: Standardized Uveitis Nomenclature
AAP: American Academy of Pediatrics
ANA: antinuclear antibody
CARRA: Childhood Arthritis and Rheumatology Research Alliance
PCORI: Patient-Centered Outcomes Research Institute
