# Supplementary material for: Knowledge about juvenile idiopathic arthritis-associated uveitis: more frequent reminders are associated with higher patient and family uveitis knowledge
Source: Pediatr Rheumatol Online J. 2021 Oct 2;19:149. doi: 10.1186/s12969-021-00639-6 (PMC8487555; doi:10.1186/s12969-021-00639-6)
Supplement: Supplementary file 1 — Additional file 1. Uveitis Education Survey Content. This document shows the detailed content of the survey utilized in this study and demonstrates the branching logic utilized to deliver appropriately worded versions of survey questions to patients versus parents. [file 12969_2021_639_MOESM1_ESM.pdf]

<u>Uveitis Education Survey Content</u>

Choose the statement that best describes you.

- I have juvenile arthritis (JIA)
- My child has juvenile arthritis (JIA)
- I do not have JIA and my child does not have JIA

Those who answered “I do not have JIA and my child does not have JIA” received a response stating “You have completed this survey. Thank you for your time”.

Remaining respondents proceeded with the survey.

1. Where are you completing this survey?
   - Rheumatology appointment
   - Ophthalmology appointment
   - Other

2. Please enter your age in years: ____________________

3. Please select your gender.
   - Male
   - Female

4. Please choose your race/ethnicity.
   - African American or Black
   - American Indian or Alaska Native
   - Asian American or Asian
   - Hispanic or Latino
   - Middle Eastern
   - Multiracial
   - Pacific Islander
   - White or Caucasian
   - Other

5. Which best describes where you live?
   - Midwest United States
   - Northeast United States
   - Southeast United States
   - Southwest United States
   - Northwest United States
   - Outside the United States

6. What is your highest level of education?
- o Some high school
- o High school graduate
- o Some college
- o Trade/technical/vocational training
- o College graduate
- o Postgraduate degree

7. Have you heard of uveitis (an eye disease that can happen in people with arthritis)?
- o Yes
- o No

The next section utilized branching logic depending on response to the initial question.

For those who answered "I have JIA"	For those who answered "My child has JIA"
8. Have you ever been told that you have uveitis?<br>o Yes<br>o No	8. Has your child ever been told that they have uveitis?<br>o Yes<br>o No
9. Who treats your arthritis?<br>o Pediatric rheumatologist/nurse practitioner/physician assistant<br>o Adult rheumatologist/nurse practitioner/physician assistant<br>o Primary care provider (pediatrician or family practitioner)<br>o Other (If other, please specify) ___________________________	9. Who treats your child's arthritis?<br>o Pediatric rheumatologist/nurse practitioner/physician assistant<br>o Adult rheumatologist/nurse practitioner/physician assistant<br>o Primary care provider (pediatrician or family practitioner)<br>o Other (If other, please specify) ___________________________
10. Have you ever had an eye exam?<br>o Yes<br>o No	10. Has your child ever had an eye exam?<br>o Yes<br>o No
11. Do you know if your eye exams are up to date?<br>o Yes<br>o No<br>o I don't know	11. Do you know if you child's eye exams are up to date?<br>o Yes<br>o No<br>o I don't know
12. How often are you supposed to get your eyes checked?<br>o Every 3-4 months<br>o Every 6 months	12. How often are you supposed to get your child's eyes checked?<br>o Every 3-4 months<br>o Every 6 months

○ Every 12 months<br>○ I don't know	○ Every 12 months<br>○ I don't know
13. At your arthritis visit, does your doctor remind you when your next eye exam is due?<br>○ Yes, at every visit<br>○ Yes, at some visits<br>○ No	13. At your child's arthritis visit, does your doctor remind you when your child's next eye exam is due?<br>○ Yes, at every visit<br>○ Yes, at some visits<br>○ No

14. Did you know that uveitis can cause cataracts (clouding of the lens)?
- ○ Yes
- ○ No

15. Did you know that uveitis can cause glaucoma (eye damage from high pressure)?
- ○ Yes
- ○ No

16. Did you know that uveitis can cause blindness?
- ○ Yes
- ○ No

17. Did you know that children with arthritis can get uveitis even when their arthritis is controlled?
- ○ Yes
- ○ No

18. Did you know that children with arthritis need eye screening even if they have no symptoms?
- ○ Yes
- ○ No

19. Did you know that children with arthritis need eye exams even if they are no longer on medications for arthritis?
- ○ Yes
- ○ No

This slit lamp is needed for a full eye exam to look for uveitis

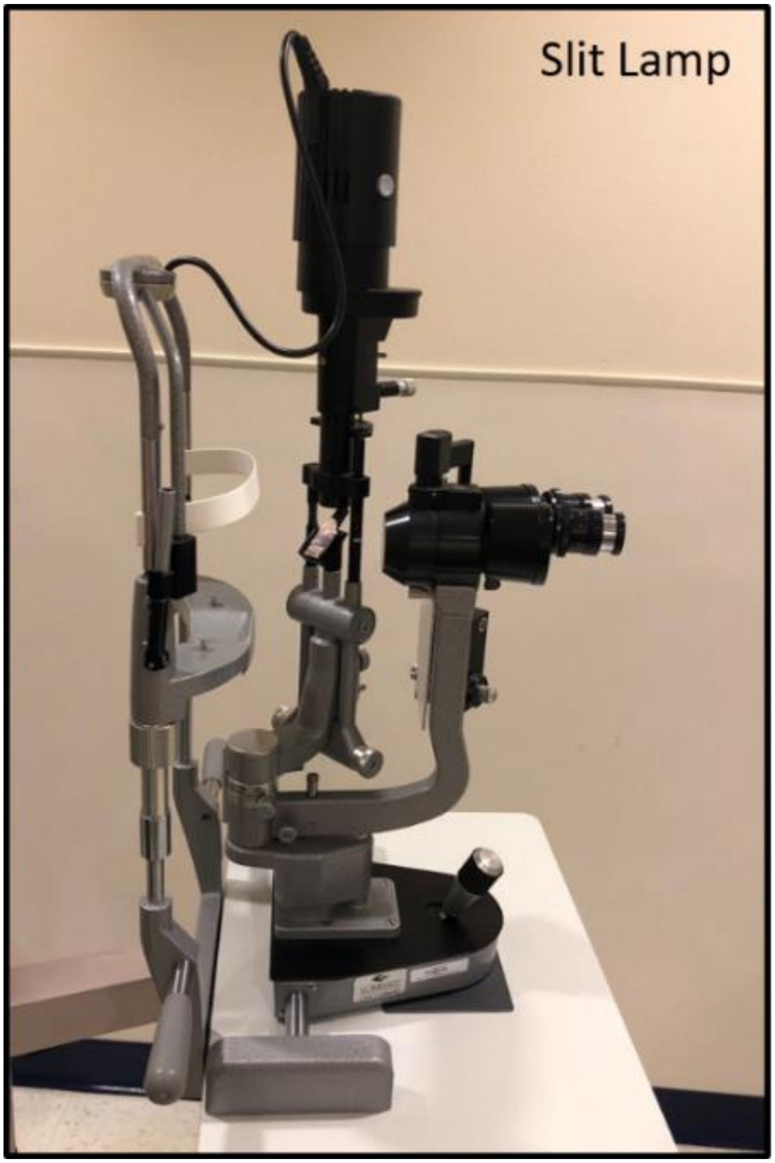

20. Did you know the slit lamp is needed for a full eye exam for uveitis?
- o Yes
- o No

An eye pressure check, as shown in this picture, is needed for a full eye exam to look for uveitis.

Examples of Tools Used to Check Eye Pressure

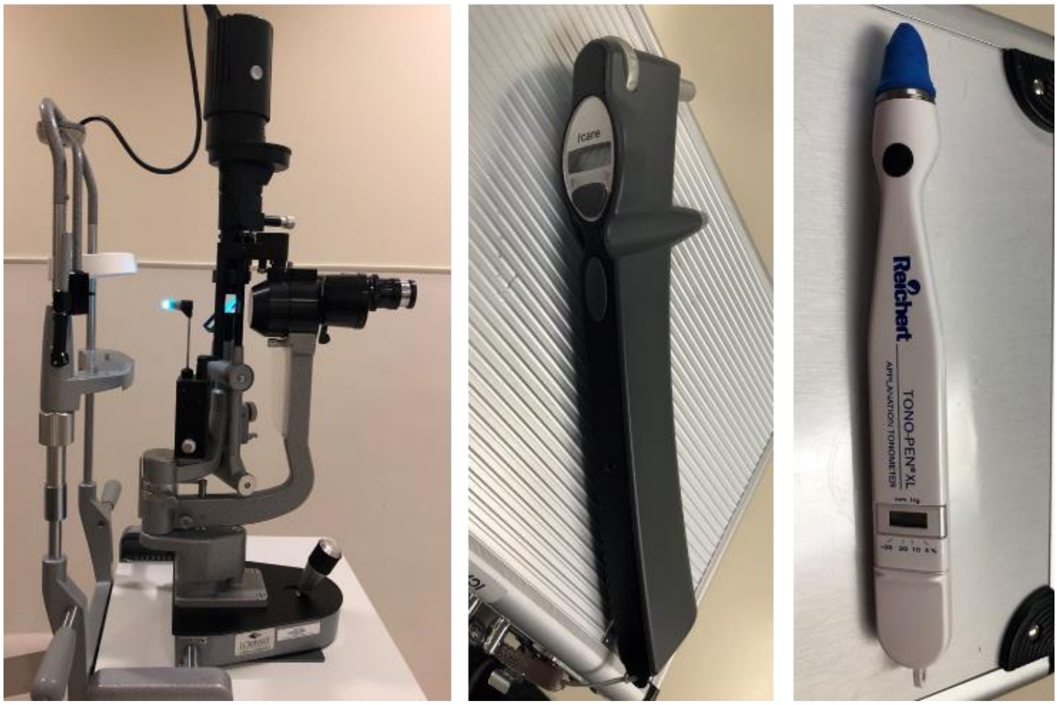

21. Did you know that an eye pressure check is needed for a full exam to look for uveitis?
- Yes
- No
